# *Chlamydia trachomatis* and *Neisseria gonorrhoeae* rectal infections: Interplay between rectal microbiome, HPV infection and Torquetenovirus

**DOI:** 10.1371/journal.pone.0301873

**Published:** 2024-04-05

**Authors:** Camilla Ceccarani, Valeria Gaspari, Sara Morselli, Marielle Ezekielle Djusse, Simona Venturoli, Tania Camboni, Marco Severgnini, Claudio Foschi, Clarissa Consolandi, Antonella Marangoni

**Affiliations:** 1 Institute of Biomedical Technologies, National Research Council, Segrate, Italy; 2 National Biodiversity Future Center S.c.a.r.l., Palermo, Italy; 3 Dermatology Unit, IRCCS Azienda Ospedaliero-Universitaria di Bologna, Bologna, Italy; 4 Department of Medical and Surgical Sciences, Section of Microbiology, Alma Mater Studiorum—University of Bologna, Bologna, Italy; 5 Microbiology Unit, IRCCS Azienda Ospedaliero-Universitaria di Bologna, Bologna, Italy; Defense Threat Reduction Agency, UNITED STATES

## Abstract

Men having sex with men (MSM) represent a key population, in which sexually transmitted rectal infections (STIs) caused by *Chlamydia trachomatis* (CT), *Neisseria gonorrhoeae* (NG) and high-risk HPV (HR-HPV) are very common and linked to significant morbidity. Investigating the anorectal microbiome associated with rectal STIs holds potential for deeper insights into the pathogenesis of these infections and the development of innovative control strategies. In this study, we explored the interplay at the rectal site between *C*. *trachomatis*, *N*. *gonorrhoeae*, HR-HPV infection, and the anorectal microbiome in a cohort of 92 MSM (47 infected by CT and/or NG *vs* 45 controls). Moreover, we assessed the presence of Torquetenovirus (TTV), a non-pathogenic endogenous virus, considered as a possible predictor of immune system activation. We found a high prevalence of HR-HPV rectal infections (61%), especially in subjects with a concurrent CT/NG rectal infection (70.2%) and in people living with HIV (84%). In addition, we observed that TTV was more prevalent in subjects with CT/NG rectal infections than in non-infected ones (70.2% vs 46.7%, respectively). The anorectal microbiome of patients infected by CT and/or NG exhibited a reduction in *Escherichia*, while the presence of TTV was significantly associated with higher levels of *Bacteroides*. We observed a positive correlation of HR-HPV types with *Escherichia* and *Corynebacterium*, and a negative correlation with the Firmicutes phylum, and with *Prevotella*, *Oscillospira*, *Sutterella*. Our findings shed light on some of the dynamics occurring within the rectal environment involving chlamydial/gonococcal infections, HPV, TTV, and the anorectal microbiome. These data could open new perspectives for the control and prevention of STIs in MSM.

## Introduction

*Chlamydia trachomatis* (CT) and *Neisseria gonorrhoeae* (NG) represent the most common cause of sexually transmitted rectal infections (STIs) among ‘men who have sex with men’ (MSM) [1–4]. In many cases, these infections exhibit no apparent symptoms, serving as a significant reservoir for further transmission. If left untreated, they can lead to several complications and sequelae [5–7].

Chlamydial and gonococcal rectal infections have been associated with an increased susceptibility to HIV acquisition [8]. In this scenario, it is well known that HIV-positive MSM are particularly prone to high-risk papillomavirus (HR-HPV) infection, a crucial factor for the development of anal neoplasia [9].

In this context, the characterization of the microbial communities of the anorectal mucosa, where chlamydial/gonococcal and HPV infections originate and proliferate, is crucial for gaining insights into the pathogenesis of these infections and for the development of innovative prevention strategies.

In a previous paper, we explored the composition of the anorectal microbiome in a cohort of MSM, both with and without rectal STIs, and categorized the results based on the type of rectal infection (no infection vs chlamydial infection vs gonorrhea), as well as the HIV status [4]. We observed a shift in the bacterial composition between patients with sexually transmitted rectal infections and controls: infected patients were characterized by a decrease in *Escherichia*, along with an increase in anaerobic genera, including *Peptoniphilus*, *Peptostreptococcus*, and *Parvimonas* [4]. The presence of HIV had a different impact on bacterial rectal communities, modifying the relative abundance of several genera, including *Gardnerella*, *Lactobacillus*, *Corynebacterium*, and *Sutterella* [4].

Furthermore, in a preliminary cross-sectional study it was found that an anal microbial community characterized by an abundance of species from the Fusobacteria phylum is linked with HIV-positive MSM who have successfully suppressed the viral load and are concurrently infected with HPV-16 [10].

While there is limited knowledge about the bacterial profiles of the anorectal microbiome in the context of chlamydial/gonococcal or HR-HPV infection, no data is currently available about the interaction between the anorectal microbiota and Torquetenovirus (TTV), a single-stranded DNA virus present in many body fluids and considered, at present, a non-pathogenic endogenous virus [11].

Recently, it has been demonstrated that TTV can be detected in the vagina of pregnant women, with its abundance being influenced by the level of activation of the immune system [12, 13].

TTV is widely distributed globally and can be transmitted through various routes, including blood, oro-fecal, respiratory, and sexual transmission [14, 15]. TTV appears to primarily replicate in T lymphocytes, and TTV viremia may serve as a simple and sensitive indicator of host immune function [12, 16]. Recently, it has been identified as a potential infectious agent involved in acute enteritis, particularly in very young and elderly individuals [17].

In an effort to contribute to the existing body of literature on the dynamics of rectal STIs and the anorectal microenvironment, this study aimed to explore the interplay between *C*. *trachomatis*, *N*. *gonorrhoeae*, HR-HPV infection, TTV, and the anorectal microbiome. In particular, a cohort of 92 MSM was divided in two groups based on the presence of a rectal CT/NG infection (47 infected subjects *vs* 45 controls) and, for each anorectal sample, we assessed (i) the positivity for HR-HPV types, (ii) the composition of the bacterial community through 16S rRNA gene sequencing, and (iii) the presence and titer of TTV using a real-time PCR assay.

## Materials and methods

### Study population

Patients were selected among a cohort of MSM, who reported engaging in condomless receptive anal sex and attended the STI Outpatients Clinic at IRCCS S. Orsola-Malpighi Hospital in Bologna (Italy) for rectal STI screening. The enrollment process is described in detail in Ceccarani et al., 2019 [4].

Exclusion criteria included: (i) age<18, (ii) antimicrobial treatments in the month prior to enrollment, (iii) presence of inflammatory bowel diseases or infectious gastroenteritis, (iv) use of enemas within three days before the sampling. Additionally, samples positive for *Mycoplasma genitalium* and HSV rectal infections, as well as specimens positive for *Treponema pallidum* chancre were further excluded from the study.

The presence of infectious gastroenteritis in symptomatic patients was excluded using microscopic (i.e., stool microscopy for protozoa and helminths), culture-based (i.e., stool cultures for pathogenic *Salmonella*, *Shigella*, *Campylobacter*, and *Yersinia* species), serological (i.e., anti-HAV IgM antibodies), or molecular approaches (i.e., stool PCR for adenovirus, rotavirus, norovirus, *Clostridium difficile* toxins, and *Escherichia coli* pathotypes).

Each patient underwent a clinical examination and the collection of an anorectal swab (E-Swab, Copan, Brescia, Italy), subsequently used for the detection of CT, NG, HPV and TTV, as well as for microbiome analysis (see specific paragraphs). Anoscopy was not performed. Information about anorectal symptoms were recorded for each patient. Clinical and microbiological data about HIV infection were registered as well, under the patient’s consent.

The study protocol was approved by the Ethics Committee of St. Orsola-Malpighi Hospital (78/2017/U/Tess) and was conducted in accordance with the Declaration of Helsinki. Written informed consent was obtained from all participants. The recruitment period of the study was from 1 October 2017 to 31 December 2022.

### Detection of chlamydial, gonococcal and HPV anorectal infection

Anorectal swabs were processed by Versant CT/GC DNA 1.0 Assay (Siemens Healthineers, Tarrytown, NY, USA), a duplex real-time PCR test detecting the presence of CT and/or NG DNA, as described in Marangoni et al. 2015 [18]. Based on the microbiological findings, eligible patients were categorized into the following groups: ‘no rectal infection’ (negative results for CT and NG) and ‘CT/NG’ (positive for rectal CT and/or NG). The same samples were further processed by Aptima HPV assay (Hologic; Rome, Italy) for the detection of HR-HPV types [19].

The presence of *M*. *genitalium* was excluded by an in-house quantitative PCR assay [7], whereas rectal HSV and *T*. *pallidum* infections were ruled out by means of a multiplex molecular approach (FTD genital ulcer, Fast Track Diagnostics, Esch sur Alzette, Luxembourg).

### TTV detection and quantification

Using the remaining DNA eluate from Versant Assay, all rectal swabs underwent testing for the presence of TTV, following previously published protocols [20, 21]. The PCR reaction mixtures, with a final volume of 25 μL, were composed of 12.5 μL of Platinum Quantitative PCR Supermix-UDG with ROX (Invitrogen, Waltham, MA, USA), 250 nM of primers, 62 nM of the probe, and 2.5 μL of the template. All PCR reactions were carried out using the following cycling conditions on a QuantStudio Real-Time PCR system (Applied Biosystems, Waltham, MA, USA): 2 minutes at 50°C, 15 seconds at 95°C, followed by 40 cycles of 15 seconds at 95°C and 60 seconds at 60°C. For the quantification of TTV, a standard curve was established using known quantities of a synthetic oligonucleotide [21]. The results were expressed as log10 DNA copies per reaction.

### Rectal microbiome composition

The hypervariable V3-V4 regions of the bacterial 16S rRNA gene were amplified from genomic DNA extracted from rectal swabs. The PCR conditions and primer sequences were obtained from the Illumina 16S Sample Preparation Guide (https://support.illumina.com/ documents/documentation/chemistry_documentation/16s/16smetagenomic-library-prep-guide-15044223-b.pdf) (Illumina, San Diego, CA, USA), with the primers originally described in Klindworth et al. 2013 [22].

Indexed libraries were prepared by pooling equimolar amounts (4 nmol/L) of samples and subsequently sequenced on the Illumina MiSeq platform using a 2 × 300 bp sequencing run, following the manufacturer’s instructions (Illumina). 16S rRNA sequences were processed according to the same methods as described in Ceccarani et al., 2019 [4]. Briefly, raw reads were merged using Pandaseq [23] and low-quality fragments (those displaying stretches of bases with a Q-score <3 for more than 25% of their length) were removed. Bioinformatic analyses were performed using the QIIME pipeline (release 1.8.0; [24]), performing the clustering into Operational Taxonomic Units (OTUs) at a 97% identity level. Taxonomic assignment was carried out using the RDP classifier against the Greengenes database (release 13.8; ftp://greengenes.microbio.me/greengenes_release/gg_13_8_otus) with a 0.5 identity threshold [25].

Biodiversity and distribution of microorganisms were assessed through both alpha and beta-diversity analyses. Alpha-diversity was quantified using various metrics such as Chao1, observed species, Shannon diversity, Good’s coverage, and Faith’s phylogenetic diversity (PD_whole_tree). Beta-diversity analysis involved comparing the microbial community structure using weighted and unweighted UniFrac distances.

### Statistical analysis

Differences in clinical and demographic parameters were assessed using the Chi-square or the Student’s t-test, employing Prism 5.02 version for Windows (GraphPad Software, San Diego, CA, USA). In all statistical analyses, a significance level of p < 0.05 was considered.

Statistical comparisons among alpha-diversity indices were conducted using a non-parametric Monte Carlo-based test involving 999 random permutations. As per the beta-diversity analysis, the "adonis" function in the R package "vegan" (version 2.0–10; [26]) was used for the PERMANOVA testing.

At each phylogenetic level, only taxa with an average relative abundance exceeding >1% in at least one of the experimental categories were considered. This approach aimed to focus on the dominant members of the rectal microbiota. Differences in the abundances of bacterial taxa among experimental groups were examined using the non-parametric Mann-Whitney U-test. To account for multiple testing, a Benjamini-Hochberg correction was applied to all the results reported. The MATLAB software (version 2008a, Natick, MA, USA) was utilized for these analyses, and an exploratory FDR threshold of <0.15 was chosen to avoid missing potentially relevant differences among bacterial groups.

A point-biserial correlation was employed to determine the relationship between bacterial taxa abundances and TTV or HPV presence in the samples [27].

## Results

### Study population

The final dataset comprised 92 rectal samples, categorized into two groups: patients with rectal CT and/or NG infection (‘CT/NG infection’, n = 47) and individuals without chlamydial/gonococcal infection (‘no infection’, n = 45).

Clinical and epidemiological information of the subjects is reported in detail in Table 1. No significant difference in the mean age of the two groups was noticed (p = 0.9), whereas patients with rectal infections were more frequently symptomatic than those without chlamydia and or gonorrhea (p<0.0001). Within the entire dataset, a total of 25 subjects (25/92; 27.2%) were people living with HIV. We noticed that rectal infections were more common in people living with HIV (64.0% vs 43.3%; p = 0.09).

**Table 1:** Characteristics of the enrolled subjects. * indicates a significant difference.

	No infection (n = 45)	CT/NG infection (n = 47)	p value
**Mean age (years ± SD)**	32.9 ± 7.8	32.6 ± 9.5	0.9
**Rectal symptoms**	0/45 (0.0%)	18/47 (38.3%)	<0.0001*
**HIV infection**	9/45 (20.0%)	16/47 (34.0%)	0.16
**HPV infection**	23/45 (51.1%)	33/47 (70.2%)	0.08
**TTV presence**	21/45 (46.7%)	33/47 (70.2%)	0.03*

Most of the people living with HIV (17; 68%) were characterized by a well-controlled infection, with undetectable or very low viral loads (< = 10 RNA copies/mL). The CD4/CD8 ratio ranged between 0.33 and 2.62, with a mean ± SD of 0.84 ± 0.48.

Furthermore, 56 subjects were positive for HR-HPV genotypes (56/92; 60.9%). CT/NG infected subjects were more likely to be positive for rectal HPV (70.2 vs 51.1%; p = 0.08), although not statistically significant. In addition, we observed a significant association between HPV and HIV status: HPV infection was significantly more frequent in people living with HIV (84.0% vs 52.2%; p = 0.007) than in HIV-free ones.

TTV was detected in 54/92 (58.6%) patients. The presence of CT/NG rectal infection was significantly associated with TTV positivity (p = 0.03), whereas no significant association was found between TTV and the presence of symptoms among patients with CT/NG rectal infections (TTV positivity in 66.6% of symptomatic patients vs 72.4% of asymptomatic subjects; p = 0.74).

TTV titers ranged between 1.5 and 8.5 log10 DNA copies per reaction. No association between TTV titer and rectal infections, as well as between TTV presence/titer and HIV, HIV viral load, or HPV was found.

### Rectal microbiome structure characterization

All the samples included in the study generated a number of reads (>17000) sufficient to have a reliable picture of the microbial composition.

**Fig 1:**
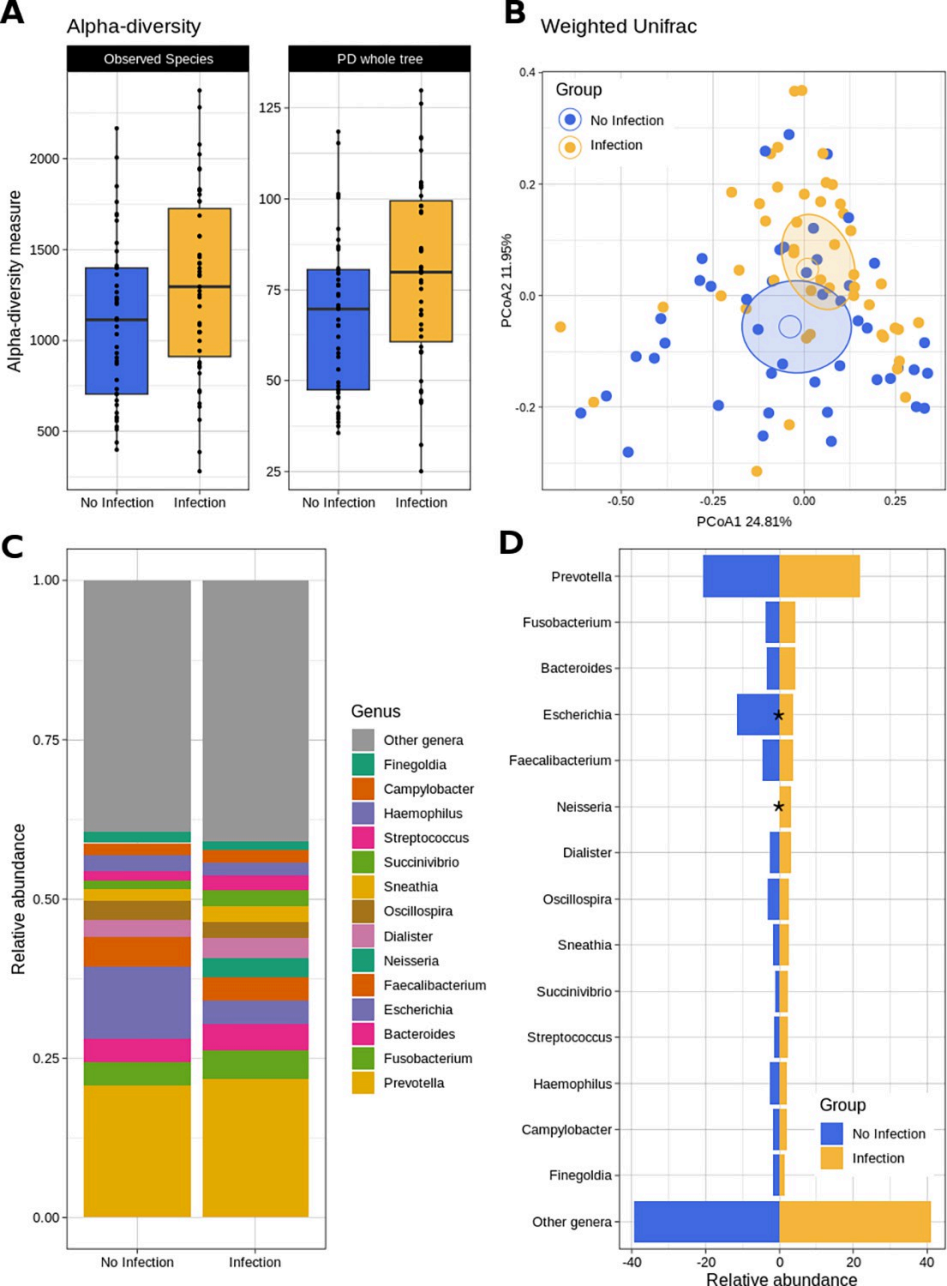
Rectal microbiota characterization (biodiversity and bacterial genera) in experimental groups: ‘infection’ (CT/NG rectal infection) vs ‘no infection’. A) Boxplot depicting alpha-diversity comparison between experimental groups for the “Observed species” (p-value = 0.03) and the “PD whole tree” (p-value = 0.019) metrics. Individual values for the 92 samples are superimposed to the plot; B) Beta-diversity Principal Coordinate Analysis (PCoA) plot derived from the weighted UniFrac distance (p-value = 0.004). Each point represents a sample, colored according to the experimental group, the centroid is the mean coordinate per group and the ellipse is the SEM-based confidence interval; C) average relative abundance of bacterial genera for the experimental groups; D) mirror plot reporting abundance proportions of genera with significant (i.e., p-value<0.05) differences highlighted by the * symbol.

Samples from CT/NG-infected patients showed a significantly higher biodiversity (alpha-diversity, p = 0.03 and p = 0.019 for the Observed species and the PD whole tree metrics, respectively), and a different microbial profile as compared to samples from non-infected patients (p = 0.003 and p = 0.004 for unweighted and weighted UniFrac distances, respectively). Significant differences were detected in the relative abundance (rel. ab.) of the genus *Escherichia* (more abundant in non-infected subjects, average rel. ab.: 11.4% vs 3.8% in non-infected and infected subjects, respectively, p = 0.022, Mann-Whitney U-test with Benjamini-Hochberg correction) and, as expected, *Neisseria* (increased in infected subjects, average rel. ab.: 0.1% vs. 2.6% in non-infected and infected subjects, respectively, p<0.001) and *Chlamydia* (<0.1% vs 0.5% in non-infected and infected subjects, p = 0.005). Moreover, we observed slight, though not statistically significant, increases in *Fusobacterium* (p = 0.116), *Succinivibrio* (p = 0.888), *Streptococcus* (p = 0.715), *Sneathia* (p = 0.540), along with a decrease of *Faecalibacterium* (p = 0.396) and *Corynebacterium* (p = 0.098) in infected patients (Fig 1).

The presence of rectal symptoms did not significantly modify the rectal microbiota profiles among the patients with CT/NG rectal infections (data not shown).

The anorectal microbiota of TTV+ and TTV- subjects *per se* was not significantly altered, neither on alpha- nor on beta-diversity (S1 Fig). However, the point biserial correlation between TTV presence/absence of the subjects and bacterial taxa revealed a significant positive correlation between TTV presence and the abundances of family *Bacteroidaceae* (0.26; p = 0.013) and genus *Bacteroides* (0.27; p = 0.009).

**Fig 2:**
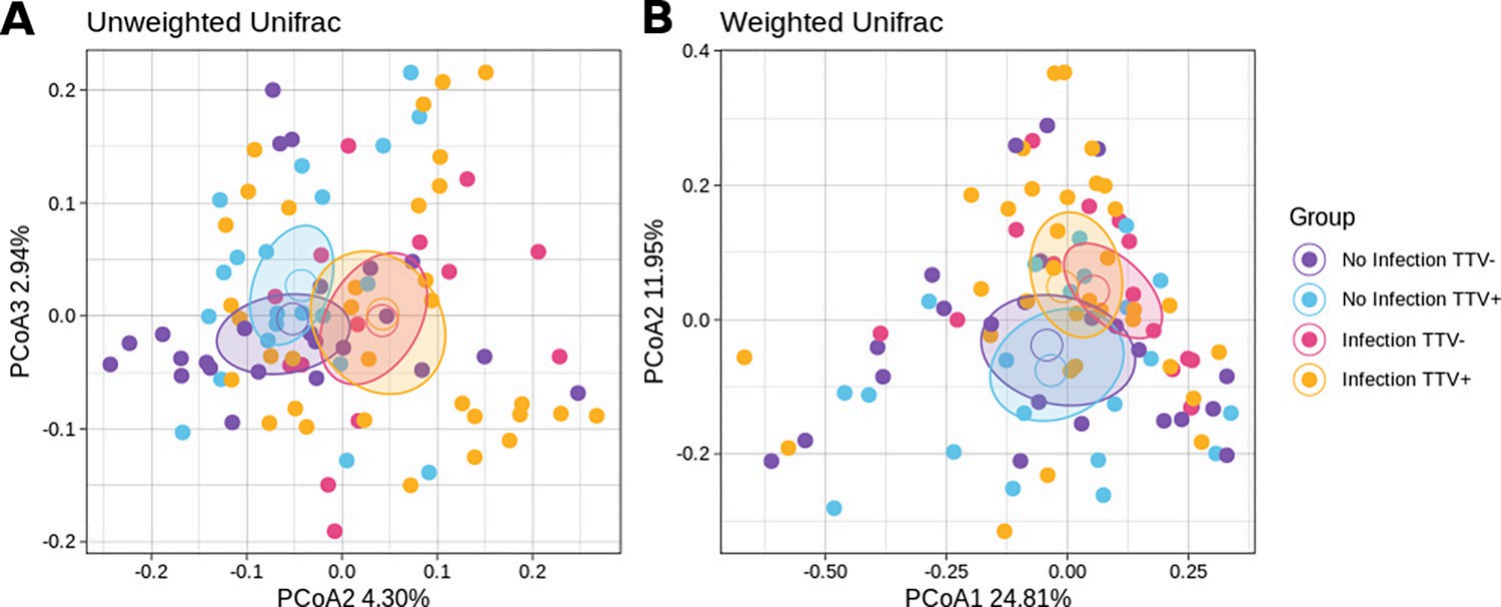
Beta-diversity PCoA plot for the combined effect of CT/NG infection and TTV presence/absence for the unweighted (A) and weighted (B) UniFrac distances among samples. Each point represents a sample, colored according to the experimental group: the centroid is the mean coordinate per group and the ellipse is the SEM-based confidence interval.

By combining experimental categories according to the presence of CT/NG infection and presence/absence of TTV, we found some interesting features. Although there was no particular trend in sample biodiversity, on the other hand, CT/NG-infected TTV+ patients resulted having a different microbial profile from non-infected ones, regardless of TTV presence (p≤0.03 vs. both TTV+ and TTV-, weighted UniFrac) (Fig 2), with *Neisseria* and *Chlamydia* abundances as the main drivers of the difference. At the same time, CT/NG infected TTV- patients did not differ significantly from non-infected ones in terms of both alpha- and beta-diversity, as well as bacterial relative abundances. The taxonomic analysis stratified by both CT/NG infection and TTV presence/absence is available as S1 Table (Supporting information).

**Fig 3:**
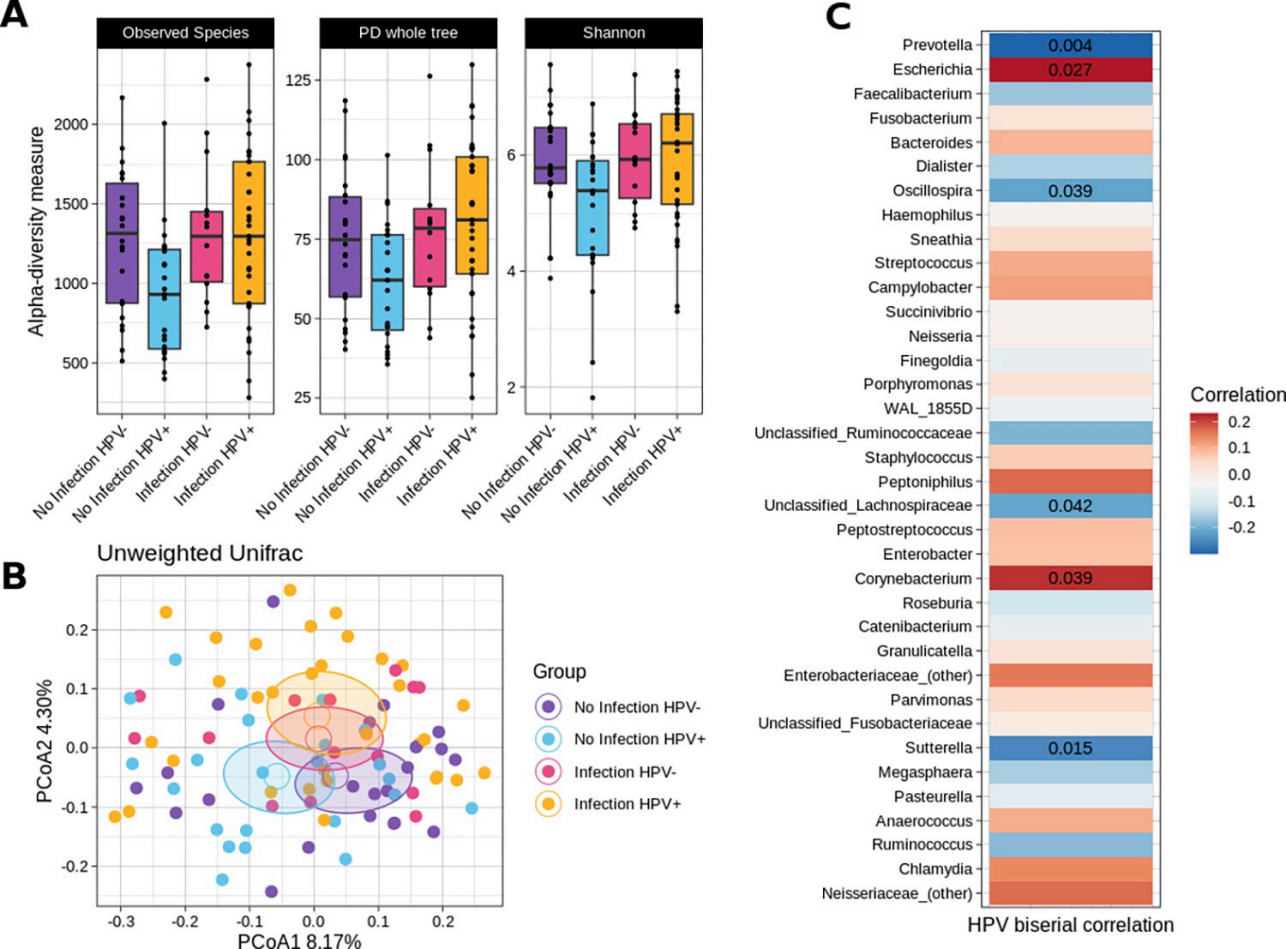
(A) Boxplot depicting alpha-diversity comparison among experimental groups (combined effect of CT/NG infection and HPV presence/absence) for the “Observed species” (p-value = 0.03), the PD whole tree (p = 0.048) and the Shannon’s diversity (p = 0.042) metrics. Individual values for the 92 samples are superimposed to the plot; (B) Beta-diversity PCoA plot for the combined effect of CT/NG infection and HPV presence/absence for the unweighted UniFrac distances among samples. Each point represents a sample, colored according to the experimental group: the centroid is the mean coordinate per group and the ellipse is the SEM-based confidence interval; (C) Point biserial correlation and p-values among HPV presence and bacterial genera in the study population. Genera are ordered based on their relative abundance values. Only significant p-values are reported. Point biserial correlation values range from 0.231 (positive correlation, red) to -0.299 (negative correlation, blue).

Similarly, presence or absence of HPV in the samples somehow influenced the rectal microbiome. Even though HPV+ and HPV- samples were not different in terms of alpha- (p-value = 0.568, PD whole tree metric) or beta-diversity (p = 0.128, unweighted UniFrac metric), stratifying the dataset on the basis of CT/NG infection and HPV presence revealed some interesting features. CT/NG-infected HPV+ patients exhibited higher alpha-diversity than CT/NG-non-infected HPV+ individuals (p<0.05 for observed species, Shannon, and PD whole tree metrics), whereas HPV- subjects were similar regardless of CT/NG infection status. Additionally, CT/NG-infected HPV+ patients showed a distinct microbial profile compared to CT/NG-non-infected subjects (irrespective of HPV+/- status) (p = 0.03 and p = 0.04 for unweighted and weighted UniFrac distances, respectively), while no significant difference was observed when compared to CT/NG-infected HPV- subjects (Fig 3A and 3B). The taxonomic analysis stratified for both CT/NG infection and HPV presence/absence is available as S2 Table (Supporting information).

HPV presence in the samples was positively correlated to *Escherichia* (0.231) and *Corynebacterium* (0.216) and negatively to several genera, such as *Prevotella* (-0.299), *Oscillospira* (-0.216), unclassified members of the *Lachnospiraceae* (-0.212), *Sutterella* (-0.252), and families, like *Veillonellaceae* (-0.226), *Alcaligenaceae* (-0.213), and *Coriobacteriaceae* (-0.231). In general, a negative correlation between HPV presence and the abundance of members of the Firmicutes phylum was observed (-0.221) (Fig 3C).

Finally, when examining individuals living with HIV, the following findings were noticed. CT/NG-infected HIV+ patients showed no differences as per the alpha-diversity analysis (p = 1 for each metric) when compared to (i) CT/NG-infected HIV-, (ii) CT/NG-non-infected HIV+, and (iii) CT/NG-non-infected HIV- subjects. On the other hand, CT/NG-infected HIV+ patients exhibited a beta-diversity profile significantly different from CT/NG-non-infected subjects (p = 0.003 and p = 0.025 for unweighted and weighted UniFrac distances, respectively, compared to CT/NG-non-infected HIV-; p = 0.021 with the weighted UniFrac distance compared to CT/NG-non-infected HIV+). Both weighted and unweighted distances also showed significant differences when comparing CT/NG-infected HIV- and CT/NG-non-infected HIV- (respectively, p = 0.006 and p = 0.029), whereas no difference was evident when comparing CT/NG-infected HIV- to CT/NG-non-infected HIV+ subjects. Detailed results are displayed in S3 Table (Supporting Information).

## Discussion

In this study, we assessed the interplay between rectal infections caused by CT/NG, the anorectal microbiome, HR-HPV infection, and TTV in a group of MSM reporting condomless rectal intercourse. To this purpose, a cohort of MSM was divided into two groups based on the rectal positivity for CT and/or NG (‘CT/NG infection’, n = 47 vs ‘no infection’, n = 45) and their rectal samples were further analyzed to assess the microbial composition, the presence of HR-HPV types and the positivity for TTV.

At first, we found that HR-HPV types are extremely frequent at the anorectal site of MSM, being the prevalence higher in subjects with concurrent CT/NG rectal infection and in people living with HIV. These results align with recent cross-sectional studies reporting a high prevalence of anal HR-HPV genotypes, especially in MSM with specific risk factors, such as the use of recreational drugs, the diagnosis of ≥2 STIs, the HIV status, and no use of condoms [28, 29].

The interplay between the risk of HPV, the HIV status, and the presence of rectal bacterial STIs has been recently discussed by Bruzzesi et al. [30]. The authors emphasized that individuals living with HIV are at a higher risk of HPV infection and that a history of gonorrhea resulted to be a risk factor at multivariable analysis, potentially creating an environment conducive to HPV entry and persistence.

Second, we observed that TTV was significantly more prevalent in subjects with CT/NG rectal infections compared to non-infected individuals. This aspect is not surprising if we consider that TTV has been recognized as a marker of immune system activation [11].

In the cervico-vaginal environment TTV is considered an indicator of the vaginal local ‘immune’ status, as its loads are related to the presence of activated lymphoid cells [21]. Similarly, we can speculate that the inflammatory response elicited by CT/NG in the anorectal mucosa is able to recruit activated lymphoid cells, which represent the preferential TTV replication site [31–33].

Interesting data emerged when looking at the composition of the rectal microbiome, stratified by the different available variables (i.e., bacterial infections, HPV positivity, TTV presence).

Consistent with previous findings [4], *Escherichia* levels were significantly lower in rectal samples from CT/NG-positive patients. However, it is challenging to ascertain whether the reduction of *Escherichia* precedes or follows CT/NG infections: this result can be interpreted in several ways. The inflammatory environment triggered by chlamydial/gonococcal infections could perturb the homeostasis of the rectal bacterial communities, leading to the proliferation/depletion of specific genera [31, 32]. In addition, the nutritional requirements of CT and NG could alter specific metabolic pathways, enabling the preferential replication of some bacterial genera compared to others [34, 35].

The presence of TTV itself did not have a significant impact on the profile of the rectal microbiome. In fact, the only notable observation was a positive correlation between TTV and the abundance of *Bacteroides* genus. However, the most pronounced alterations of the rectal microbiome (as resulting from the beta-diversity analysis based on the weighted UniFrac distance) were observed when CT/NG infection occurred with TTV. Thus, future studies are needed to better evaluate the impact of TTV on the rectal microbial and immune environment and whether other variables (e.g., HIV status, frequency of receptive anal sex, use of douching/enemas) influence the presence and activity of this endogenous virus.

When examining the impact of HR-HPV types, we observed that the presence of HPV per se did not significantly modify the structure of the anorectal microbiome (no differences in terms of alpha- or beta-diversity). However, it is worth noting that HPV could amplify microbial diversity among patients with or without CT/NG rectal infections, potentially acting as a significant co-factor in driving the microbial changes of the anorectal community.

Previous studies have mainly investigated the impact of HPV on the cervico-vaginal microbiome, demonstrating that high levels of *Lactobacillus crispatus* are associated with decreased HPV detection and that *Gardnerella* is the dominant biomarker for HR-HPV progression [36, 37].

In contrast, limited data are available regarding the effect of HPV on the anorectal microbiome profile [10, 38, 39]. Overall, these works highlight specific microbial fingerprints of the anorectal microbiome indicative of HR-HPV infection or precancerous lesions. For example, Ron and colleagues detected 40 potential predictors of biopsy-proven high-grade intraepithelial squamous neoplasia (HSIL), including members of *Ruminococcaceae* group, *Alloprevotella* genus, and *Prevotella melaninogenica* [39]. Nowak et al. suggested that a higher proportion of MSM with prevalent HPV-16 are associated with a cluster of the ano-rectal microbiome enriched with *Sneathia*, a bacterium from the family of *Fusobacteriaceae* [10].

The depletion of *Prevotella* and of *Oscillospira* (a Firmicutes member from the *Ruminococcaceae* family) found in our dataset of HPV-positive MSM appears to differ from the findings of Ron et al. [39]. However, it should be underlined that we only detected the presence of HR-HPV types by NAAT, with no information about the presence and progression of HPV-related lesions. Presumably, each stage of HPV infection could be associated with a distinct microbial signature in the anorectal microbiome.

We are fully aware of some limitations of this study. First, this is a cross-sectional study with no sampling during the follow-up period; thus, further prospective investigations are needed to understand if the microbiome alterations are a risk factor or a consequence of the rectal infections (i.e., HPV and chlamydia/gonorrhea). Second, the lack of additional clinical/behavioral information (e.g., number of sexual partners, HPV vaccine, presence of anal precancerous lesions) has limited the ability to establish deeper correlations between HPV, TTV, rectal STIs and the microbiome alterations.

Our findings contribute to expanding the knowledge on the interactions between pathogens responsible for rectal STIs and the anorectal environment, being potentially useful to set up innovative strategies in the prevention and control of these infections. These data could open new perspectives in the diagnostic/prognostic approaches of rectal STIs, as (i) the characterization of the rectal microbiome to evaluate the risk of STI acquisition and the progression of HPV infection/lesions, (ii) the detection of TTV as a marker of immune system activation, and its correlation with the extent of rectal inflammation.

## Supporting information

S1 Fig (A) Boxplots depicting alpha-diversity comparison between TTV+ and TTV- subjects for all the alpha-diversity metrics. Individual values for the 92 samples are superimposed to the plot; (B) Unweighted and (C) Weighted PCoA plots. Each point represents a sample, colored according to the TTV presence: the centroid is the mean coordinate per group and the ellipse is the SEM-based confidence interval.
(TIF)

S1 Table Taxonomic relative abundances at genus level for infected and not infected patients divided by their positivity to TTV. For all bacterial genera that are present in at least 1% in any experimental category, data are reported as mean (SD); significant adjusted p-values (i.e., p<0.05) are underlined.
(DOCX)

S2 Table Taxonomic relative abundances at genus level for infected and not infected patients divided by their positivity to HPV. For all bacterial genera that are present in at least 1% in any experimental category, data are reported as mean (SD); significant adjusted p-values (i.e., p<0.05) are underlined.
(DOCX)

S3 Table Taxonomic relative abundances at genus level for infected and not infected patients divided by their positivity for HIV. For all bacterial genera that are present in at least 1% in any experimental category, data are reported as mean (SD); significant adjusted p-values (i.e., p<0.05) are underlined.
(DOCX)
