# Supplementary figures and images for: Chlamydia trachomatis and Neisseria gonorrhoeae rectal infections: Interplay between rectal microbiome, HPV infection and Torquetenovirus
Source: PLoS One. 2024 Apr 5;19(4):e0301873. doi: 10.1371/journal.pone.0301873 (PMC10997096; doi:10.1371/journal.pone.0301873)

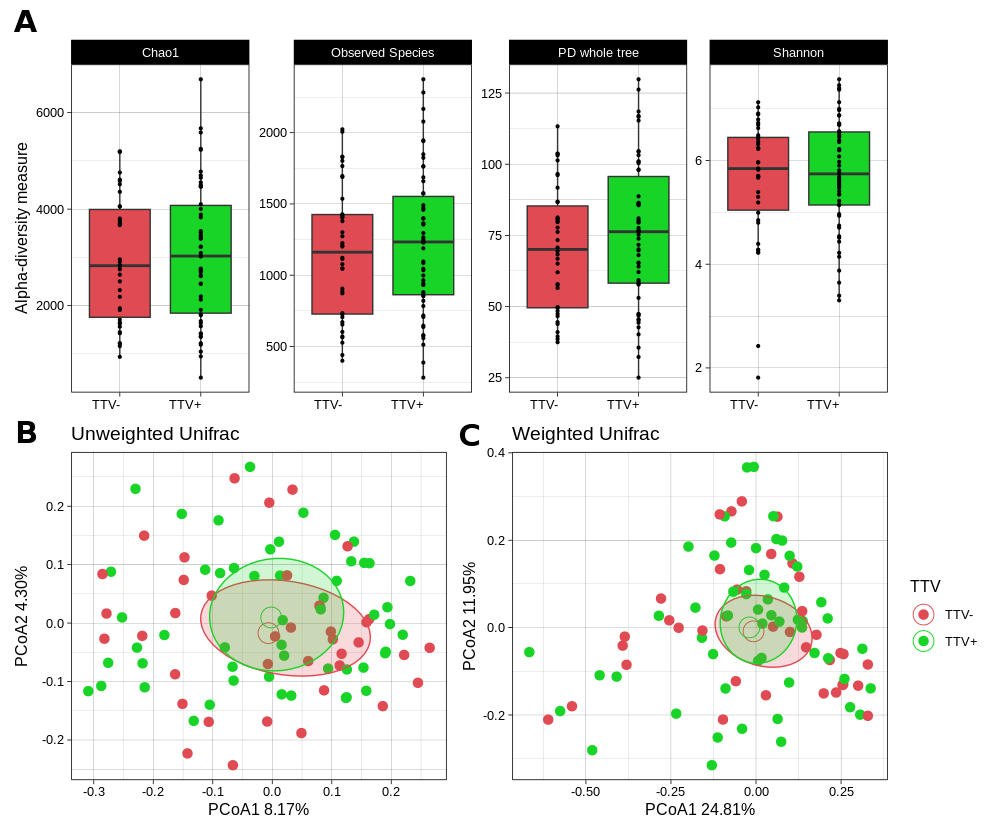

Supplement: S1 Fig — (A) Boxplots depicting alpha-diversity comparison between TTV+ and TTV- subjects for all the alpha-diversity metrics. Individual values for the 92 samples are superimposed to the plot; (B) Unweighted and (C) Weighted PCoA plots. Each point represents a sample, colored according to the TTV presence: the centroid is the mean coordinate per group and the ellipse is the SEM-based confidence interval. (TIF) [file pone.0301873.s001.tif]
