# Supplementary material for: Chlamydia trachomatis and Neisseria gonorrhoeae rectal infections: Interplay between rectal microbiome, HPV infection and Torquetenovirus
Source: PLoS One. 2024 Apr 5;19(4):e0301873. doi: 10.1371/journal.pone.0301873 (PMC10997096; doi:10.1371/journal.pone.0301873)
Supplement: S1 Table — For all bacterial genera that are present in at least 1% in any experimental category, data are reported as mean (SD); significant adjusted p-values (i.e., p<0.05) are underlined. (DOCX) [file pone.0301873.s002.docx]

| **Genus** | *mean (SD)* | | | | *p-value* | | | | | |
| --- | --- | --- | --- | --- | --- | --- | --- | --- | --- | --- |
|  | **No Infection TTV-** | **No Infection TTV+** | **Infection TTV-** | **Infection TTV+** | **No Infection TTV- / No Infection TTV+** | **No Infection TTV- / Infection TTV-** | **No Infection TTV- / Infection TTV+** | **No Infection TTV+ / Infection TTV-** | **No Infection TTV+ / Infection TTV+** | **Infection TTV- / Infection TTV+** |
| *Prevotella* | 21.42 (10.48) | 20.07 (12.51) | 23.62 (11.39) | 21.04 (9.02) | 1 | 1 | 1 | 0.602 | 1 | 1 |
| *Escherichia* | 11.96 (20.94) | 10.74 (13.78) | 3.76 (6.91) | 3.80 (6.97) | 1 | 0.174 | 0.058 | 0.192 | 0.072 | 1 |
| *Fusobacterium* | 4.33 (7.68) | 2.89 (2.72) | 3.63 (3.46) | 4.68 (5.69) | 0.825 | 0.58 | 0.189 | 1 | 1 | 1 |
| *Faecalibacterium* | 3.99 (4.02) | 5.34 (5.78) | 3.59 (3.52) | 3.60 (3.65) | 1 | 1 | 1 | 1 | 0.723 | 1 |
| *Bacteroides* | 2.36 (4.21) | 4.88 (3.44) | 2.19 (3.87) | 4.97 (2.40) | 0.105 | 1 | 0.114 | 0.276 | 1 | 0.333 |
| *Dialister* | 2.69 (7.64) | 2.61 (3.20) | 3.70 (0.79) | 2.78 (3.65) | 1 | 0.227 | 1 | 0.239 | 1 | 0.592 |
| *Oscillospira* | 3.19 (2.99) | 2.91 (2.42) | 3.23 (2.18) | 2.34 (2.02) | 1 | 1 | 1 | 1 | 1 | 1 |
| *Succinivibrio* | 1.36 (3.82) | 1.27 (4.96) | 2.55 (2.73) | 2.34 (5.81) | 1 | 1 | 1 | 1 | 1 | 1 |
| *Streptococcus* | 0.93 (5.70) | 2.17 (4.43) | 1.83 (3.89) | 2.58 (4.47) | 0.868 | 1 | 1 | 1 | 1 | 1 |
| *Sneathia* | 2.14 (4.22) | 1.42 (6.77) | 2.11 (0.81) | 2.63 (7.73) | 1 | 1 | 1 | 1 | 1 | 1 |
| *Finegoldia* | 1.60 (4.84) | 1.94 (4.05) | 2.28 (2.50) | 1.00 (0.49) | 1 | 0.804 | 1 | 1 | 1 | 0.752 |
| *Peptoniphilus* | 1.04 (5.46) | 0.62 (3.44) | 2.33 (0.01) | 1.50 (0.54) | 1 | 0.025 | 0.217 | 0.017 | 0.145 | 0.617 |
| *Porphyromonas* | 1.16 (2.76) | 0.70 (3.34) | 1.67 (1.66) | 2.12 (2.35) | 1 | 0.672 | 1 | 0.376 | 1 | 1 |
| *Peptostreptococcus* | 0.60 (6.02) | 0.77 (3.83) | 2.29 (8.87) | 1.37 (0.87) | 0.895 | 0.001 | 0.003 | 0.034 | 0.131 | 0.987 |
| *Granulicatella* | 0.53 (2.54) | 0.57 (2.17) | 1.75 (2.75) | 0.82 (1.10) | 1 | 0.106 | 0.392 | 0.261 | 0.908 | 1 |
| *Megasphaera* | 0.87 (1.51) | 0.67 (1.26) | 0.73 (0.83) | 0.75 (0.97) | 1 | 1 | 1 | 1 | 1 | 1 |
| *Corynebacterium* | 1.71 (1.62) | 1.38 (2.02) | 1.13 (1.34) | 0.32 (1.55) | 1 | 0.648 | 0.197 | 1 | 1 | 1 |
| *Enterobacter* | 1.65 (3.41) | 1.08 (3.25) | 2.48 (4.45) | 0.16 (3.59) | 1 | 1 | 0.173 | 0.883 | 0.074 | 1 |
| *Staphylococcus* | 2.76 (1.64) | 1.22 (0.96) | 0.27 (1.13) | 0.92 (1.24) | 1 | 0.903 | 0.156 | 1 | 0.386 | 1 |
| *Haemophilus* | 2.02 (1.81) | 3.02 (1.42) | 0.35 (1.48) | 2.71 (0.91) | 0.91 | 0.83 | 1 | 0.153 | 1 | 0.424 |
| *Neisseria* | 0.01 (1.99) | 0.13 (1.07) | 1.27 (0.66) | 3.87 (1.34) | 0.982 | 0.056 | 0.002 | 0.446 | 0.073 | 1 |
| *Parvimonas* | 0.64 (2.08) | 0.29 (1.45) | 1.31 (3.17) | 1.07 (3.41) | 1 | 0.038 | 0.138 | 0.09 | 0.327 | 1 |
| *Anaerococcus* | 0.79 (2.30) | 0.45 (1.67) | 1.25 (3.34) | 0.46 (3.01) | 0.856 | 0.286 | 1 | 0.032 | 1 | 0.164 |
| *Pasteurella* | 1.68 (1.36) | 1.06 (0.75) | 0.00 (0.59) | 0.16 (0.99) | 1 | 0.044 | 0.752 | 0.072 | 1 | 0.324 |
| *Ruminococcus* | 0.77 (1.58) | 0.64 (0.65) | 0.65 (2.26) | 0.59 (1.79) | 1 | 1 | 1 | 1 | 1 | 1 |
| *Unclassified_Ruminococcaceae* | 1.51 (3.99) | 1.48 (0.06) | 1.32 (0.01) | 1.26 (0.03) | 1 | 1 | 1 | 1 | 1 | 1 |
| *Chlamydia* | 0.01 (1.43) | 0.01 (3.26) | 0.13 (3.61) | 0.96 (5.26) | 1 | 0.709 | 0.015 | 1 | 0.05 | 0.805 |

**S1 Table**. Taxonomic relative abundances at genus level for infected and not infected patients divided by their positivity to TTV. For all bacterial genera that are present in at least 1% in any experimental category, data are reported as mean (SD); significant adjusted p-values (i.e., p<0.05) are underlined.
