# Supplementary material for: Chlamydia trachomatis and Neisseria gonorrhoeae rectal infections: Interplay between rectal microbiome, HPV infection and Torquetenovirus
Source: PLoS One. 2024 Apr 5;19(4):e0301873. doi: 10.1371/journal.pone.0301873 (PMC10997096; doi:10.1371/journal.pone.0301873)
Supplement: S2 Table — For all bacterial genera that are present in at least 1% in any experimental category, data are reported as mean (SD); significant adjusted p-values (i.e., p<0.05) are underlined. (DOCX) [file pone.0301873.s003.docx]

| **Genus** | *mean (SD)* | | | | *p-value* | | | | | |
| --- | --- | --- | --- | --- | --- | --- | --- | --- | --- | --- |
|  | **No Infection HPV-** | **No Infection HPV+** | **Infection HPV-** | **Infection HPV+** | **No Infection HPV- / No Infection HPV+** | **No Infection HPV- / Infection HPV-** | **No Infection HPV- / Infection HPV+** | **No Infection HPV+ / Infection HPV-** | **No Infection HPV+ / Infection HPV+** | **Infection HPV- / Infection HPV+** |
| *Prevotella* | 22.72 (11.91) | 18.94 (10.73) | 25.4 (10.31) | 20.28 (9.21) | 0.56 | 0.982 | 1 | 0.095 | 1 | 0.263832007 |
| *Escherichia* | 7.23 (11.31) | 15.37 (21.81) | 2.73 (4.10) | 4.23 (7.77) | 0.615 | 0.637 | 0.322 | 0.053 | 0.008 | 1 |
| *Fusobacterium* | 2.98 (4.09) | 4.31 (7.26) | 2.89 (2.97) | 4.99 (5.71) | 1 | 1 | 0.308 | 1 | 0.46 | 1 |
| *Faecalibacterium* | 6.07 (5.87) | 3.24 (3.35) | 3.28 (2.75) | 3.73 (3.90) | 0.374 | 0.716 | 0.391 | 1 | 1 | 1 |
| *Bacteroides* | 4.62 (5.55) | 2.51 (3.02) | 4.81 (6.69) | 3.86 (4.55) | 1 | 1 | 1 | 1 | 0.813 | 1 |
| *Dialister* | 3.33 (3.19) | 2.01 (2.02) | 3.17 (1.78) | 3.00 (2.23) | 0.166 | 1 | 1 | 0.085 | 0.157 | 1 |
| *Oscillospira* | 3.63 (3.72) | 2.52 (3.94) | 2.70 (2.91) | 2.57 (2.94) | 0.543 | 1 | 1 | 1 | 1 | 1 |
| *Succinivibrio* | 1.59 (4.21) | 1.05 (2.16) | 2.58 (4.06) | 2.32 (3.77) | 1 | 1 | 1 | 1 | 1 | 1 |
| *Streptococcus* | 0.89 (1.61) | 2.10 (3.06) | 2.51 (4.10) | 2.30 (5.12) | 0.9 | 0.608 | 1 | 1 | 1 | 1 |
| *Sneathia* | 1.10 (3.44) | 2.48 (6.31) | 1.79 (3.72) | 2.77 (4.51) | 1 | 1 | 0.944 | 1 | 1 | 1 |
| *Finegoldia* | 1.83 (2.05) | 1.69 (2.66) | 1.94 (2.03) | 1.14 (1.71) | 1 | 0.775 | 0.846 | 0.233 | 1 | 0.09702224 |
| *Peptoniphilus* | 0.72 (1.08) | 0.96 (1.39) | 1.44 (2.01) | 1.88 (1.95) | 1 | 0.233 | 0.032 | 0.358 | 0.063 | 1 |
| *Porphyromonas* | 0.77 (1.29) | 1.11 (2.22) | 0.56 (1.11) | 2.60 (3.74) | 1 | 1 | 0.431 | 1 | 0.285 | 0.293444825 |
| *Peptostreptococcus* | 0.49 (1.06) | 0.86 (1.30) | 2.09 (2.11) | 1.45 (1.48) | 0.485 | 0.001 | 0.001 | 0.067 | 0.127 | 1 |
| *Granulicatella* | 0.45 (0.88) | 0.64 (1.13) | 2.34 (3.18) | 0.57 (1.22) | 1 | 0.013 | 0.584 | 0.065 | 1 | 0.159039543 |
| *Megasphaera* | 0.91 (1.32) | 0.65 (1.03) | 1.28 (1.41) | 0.51 (0.72) | 1 | 1 | 1 | 0.881 | 1 | 0.341017646 |
| *Corynebacterium* | 0.79 (1.37) | 2.30 (6.05) | 0.96 (2.31) | 0.40 (0.85) | 1 | 1 | 0.569 | 1 | 0.069 | 0.376191688 |
| *Enterobacter* | 1.38 (6.09) | 1.39 (4.00) | 2.48 (8.87) | 0.17 (0.87) | 1 | 1 | 0.182 | 0.969 | 0.065 | 1 |
| *Staphylococcus* | 0.94 (2.87) | 3.10 (7.83) | 1.54 (5.33) | 0.38 (1.34) | 0.495 | 1 | 0.461 | 1 | 0.009 | 0.174452479 |
| *Haemophilus* | 1.83 (4.45) | 3.11 (6.40) | 1.50 (2.40) | 2.23 (7.69) | 1 | 1 | 1 | 1 | 0.64 | 1 |
| *Neisseria* | 0.12 (0.54) | 0.02 (0.06) | 1.95 (4.77) | 3.59 (9.12) | 1 | 0.152 | 0.01 | 0.185 | 0.014 | 1 |
| *Parvimonas* | 0.24 (0.48) | 0.70 (1.58) | 0.71 (0.88) | 1.33 (2.27) | 0.311 | 0.259 | 0.002 | 1 | 0.291 | 0.818523509 |
| *Anaerococcus* | 0.42 (0.45) | 0.82 (1.24) | 0.66 (0.80) | 0.71 (0.77) | 1 | 0.886 | 0.805 | 1 | 1 | 1 |
| *Pasteurella* | 0.97 (2.20) | 1.80 (6.10) | 0.04 (0.07) | 0.15 (0.54) | 1 | 0.527 | 0.117 | 1 | 1 | 1 |
| *Ruminococcus* | 1.02 (1.22) | 0.40 (0.69) | 0.47 (0.94) | 0.67 (0.94) | 0.086 | 0.202 | 0.657 | 1 | 0.742 | 1 |
| *Unclassified_Ruminococcaceae* | 2.06 (2.07) | 0.96 (1.33) | 1.29 (1.35) | 1.27 (1.55) | 0.163 | 1 | 0.521 | 1 | 1 | 1 |
| *Chlamydia* | 0.01 (0.02) | 0.01 (0.04) | 0.15 (0.28) | 0.95 (1.99) | 1 | 0.127 | 0.035 | 0.367 | 0.16 | 1 |

**S2 Table**. Taxonomic relative abundances at genus level for infected and not infected patients divided by their positivity to HPV. For all bacterial genera that are present in at least 1% in any experimental category, data are reported as mean (SD); significant adjusted p-values (i.e., p<0.05) are underlined.
