# Supplementary material for: Chlamydia trachomatis and Neisseria gonorrhoeae rectal infections: Interplay between rectal microbiome, HPV infection and Torquetenovirus
Source: PLoS One. 2024 Apr 5;19(4):e0301873. doi: 10.1371/journal.pone.0301873 (PMC10997096; doi:10.1371/journal.pone.0301873)
Supplement: S3 Table — For all bacterial genera that are present in at least 1% in any experimental category, data are reported as mean (SD); significant adjusted p-values (i.e., p<0.05) are underlined. (DOCX) [file pone.0301873.s004.docx]

|  | **No Infection HIV-** | | **No Infection HIV+** | | **Infection HIV-** | | **Infection HIV+** | | **Infection HIV-** | **Infection HIV-** | **Infection HIV-** | **Infection HIV+** | **Infection HIV+** | **No Infection HIV-** |
| --- | --- | --- | --- | --- | --- | --- | --- | --- | --- | --- | --- | --- | --- | --- |
|  |  |  |  |  |  |  |  |  | **vs** | **vs** | **vs** | **vs** | **vs** | **vs** |
| **Genus** | **Mean** | **SD** | **Mean** | **SD** | **Mean** | **SD** | **Mean** | **SD** | **Infection HIV+** | **No Infection HIV-** | **No Infection HIV+** | **No Infection HIV-** | **No Infection HIV+** | **No Infection HIV+** |
| *Prevotella* | 20.24 | 10.85 | 21.86 | 13.26 | 24.75 | 8.74 | 16.65 | 9.59 | 0.043 | 0.263 | 1 | 1 | 1 | 1 |
| *Escherichia* | 10.29 | 15.05 | 18.00 | 26.82 | 3.24 | 6.61 | 3.90 | 6.48 | 1 | 0.006 | 0.148 | 0.581 | 1 | 1 |
| *Faecalibacterium* | 4.76 | 5.12 | 2.83 | 2.62 | 4.11 | 4.02 | 2.93 | 2.87 | 1 | 1 | 1 | 1 | 1 | 1 |
| *Fusobacterium* | 4.15 | 6.54 | 2.29 | 3.17 | 2.85 | 3.84 | 5.88 | 5.86 | 0.183 | 1 | 1 | 0.229 | 0.256 | 1 |
| *Bacteroides* | 3.65 | 4.71 | 2.20 | 3.08 | 4.03 | 5.27 | 3.58 | 3.53 | 1 | 1 | 1 | 1 | 1 | 1 |
| *Oscillospira* | 3.22 | 3.97 | 1.17 | 1.59 | 2.49 | 2.99 | 2.53 | 2.70 | 1 | 1 | 1 | 1 | 1 | 0.909 |
| *Haemophilus* | 3.10 | 6.19 | 0.72 | 1.59 | 2.29 | 7.89 | 2.12 | 5.16 | 0.351 | 0.417 | 1 | 1 | 1 | 1 |
| *Dialister* | 2.99 | 2.92 | 1.93 | 1.58 | 3.22 | 2.12 | 2.47 | 2.12 | 0.903 | 1 | 0.433 | 1 | 1 | 1 |
| *Campylobacter* | 2.33 | 3.35 | 0.55 | 0.64 | 1.99 | 2.16 | 1.88 | 2.33 | 1 | 1 | 1 | 1 | 1 | 1 |
| *Sneathia* | 2.21 | 5.79 | 0.67 | 1.46 | 2.28 | 3.91 | 3.56 | 5.22 | 0.305 | 1 | 1 | 0.344 | 0.358 | 1 |
| *Finegoldia* | 1.88 | 2.50 | 1.63 | 2.00 | 1.55 | 1.90 | 0.92 | 1.11 | 0.599 | 1 | 1 | 1 | 1 | 1 |
| *Staphylococcus* | 1.46 | 3.84 | 4.56 | 11.26 | 0.37 | 1.41 | 1.35 | 4.99 | 1 | 0.626 | 1 | 0.658 | 1 | 1 |
| *Corynebacterium* | 1.42 | 4.13 | 2.40 | 6.08 | 0.82 | 1.88 | 0.24 | 0.41 | 0.261 | 1 | 1 | 0.083 | 0.402 | 1 |
| *Pasteurella* | 1.36 | 4.65 | 1.72 | 5.14 | 0.09 | 0.33 | 0.02 | 0.03 | 0.699 | 0.053 | 1 | 1 | 1 | 1 |
| *[Prevotella]* | 1.33 | 1.81 | 0.76 | 0.66 | 0.77 | 1.05 | 1.10 | 1.01 | 0.325 | 0.846 | 1 | 1 | 1 | 1 |
| *Enterobacter* | 1.29 | 5.07 | 1.97 | 5.86 | 0.06 | 0.24 | 2.41 | 8.32 | 1 | 0.210 | 1 | 1 | 1 | 1 |
| *Streptococcus* | 1.28 | 2.18 | 2.38 | 3.68 | 2.68 | 5.64 | 2.40 | 4.03 | 1 | 1 | 1 | 1 | 1 | 1 |
| *Succinivibrio* | 1.22 | 3.44 | 1.97 | 3.19 | 1.57 | 2.83 | 2.70 | 4.25 | 0.825 | 1 | 1 | 1 | 1 | 1 |
| *Roseburia* | 1.15 | 1.18 | 0.30 | 0.34 | 1.11 | 1.35 | 0.72 | 1.02 | 1 | 1 | 1 | 1 | 1 | 0.446 |
| *Catenibacterium* | 1.05 | 1.76 | 1.19 | 1.17 | 0.78 | 1.30 | 0.93 | 1.17 | 1 | 1 | 1 | 1 | 1 | 1 |
| *Veillonella* | 0.92 | 4.13 | 0.19 | 0.31 | 0.38 | 1.52 | 0.23 | 0.42 | 0.219 | 0.221 | 1 | 1 | 1 | 1 |
| Other genera | 28.69 |  | 28.72 |  | 38.58 |  | 41.50 |  |  |  |  |  |  |  |

**S3 Table.** Taxonomic relative abundances at genus level for infected and not infected patients divided by their positivity for HIV. For all bacterial genera that are present in at least 1% in any experimental category, data are reported as mean (SD); significant adjusted p-values (i.e., p<0.05) are underlined.
